# Lay attitudes toward deception in medicine: Theoretical considerations and empirical evidence

**DOI:** 10.1080/23294515.2015.1021494

**Published:** 2015-11-16

**Authors:** Jonathan Pugh, Guy Kahane, Hannah Maslen, Julian Savulescu

**Affiliations:** ^a^ Oxford Uehiro Centre for Practdical Ethics, University of Oxford

**Keywords:** autonomy, clinical ethics, deception, placebo

## Abstract

**Background:** There is a lack of empirical data on lay attitudes toward different sorts of deception in medicine. However, lay attitudes toward deception should be taken into account when we consider whether deception is ever permissible in a medical context. The objective of this study was to examine lay attitudes of U.S. citizens toward different sorts of deception across different medical contexts. **Methods**: A one-time online survey was administered to U.S. users of the Amazon “Mechanical Turk” website. Participants were asked to answer questions regarding a series of vignettes depicting different sorts of deception in medical care, as well as a question regarding their general attitudes toward truth-telling. **Results:** Of the 200 respondents, the majority found the use of placebos in different contexts to be acceptable following partial disclosure but found it to be unacceptable if it involved outright lying. Also, 55.5% of respondents supported the use of sham surgery in clinical research, although 55% claimed that it would be unacceptable to deceive patients in this research, even if this would improve the quality of the data from the study. Respondents supported fully informing patients about distressing medical information in different contexts, especially when the patient is suffering from a chronic condition. In addition, 42.5% of respondents believed that it is worse to deceive someone by providing the person with false information than it is to do so by giving the person true information that is likely to lead them to form a false belief, without telling them other important information that shows it to be false. However, 41.5% believed that the two methods of deception were morally equivalent. **Conclusions**: Respondents believed that some forms of deception were acceptable in some circumstances. While the majority of our respondents opposed outright lying in medical contexts, they were prepared to support partial disclosure and the use of placebos when it is in the patient's interests or when it is what the person would want. These results support the position that physicians should be allowed a greater degree of authority to make a professional judgment about whether deception might be morally warranted by the circumstances, provided that it doesn't involve outright lying.

It is all but universally agreed that a fundamental tenet of medical ethics is that physicians should not deceive their patients. For instance, the American Medical Association (AMA) seems to endorse a strong deontological principle that prohibits deception in medicine, stating in its principles of medical ethics that “A physician shall be honest in all professional interactions, and strive to report physicians engaging in fraud or deception” (2001). This view is often understood to be rooted in the principle of respect for autonomy; deception is held to undermine a patient's autonomy, treating her merely as a means (Beauchamp and Childress 2009).

There is, however, a range of medical contexts where deception would appear to be in a patient's best interests. Indeed, studies have suggested that at least some physicians believe that it may be acceptable to deceive a patient if that is in the patient's best interests, either by lying to the patient or by omitting material information in disclosures to the patient, in the context of palliative care (Bruera et al. 2000); when resolving difficult ethical problems (Novack et al. 1989); and in some cases of placebo prescription (Howick et al. 2013). Moreover, it is likely that many physicians are putting these beliefs into practice. Howick and colleagues (2013) found that 97% of UK general practitioners (GPs) had prescribed a placebo at least once in their careers, and of these, only 8% told their patients that they were being prescribed a placebo rather than an active therapy.

In these cases, two fundamental principles of medical ethics—the principle of beneficence and the principle of respect for autonomy—appear to conflict (Beauchamp and Childress 2009). While ethicists have long been interested in the conflict between these two principles in cases of deception in medical practice, there is comparatively little empirical evidence concerning whether lay people—the potential targets of such deception—regard deception as morally acceptable across different medical contexts. Empirical studies that have been carried out thus far have concerned patient attitudes toward deception in specific medical contexts, such as cancer treatment (Jenkins, Fallowfield, and Saul, 2001; Yu and Bernstein 2011), palliative care, (Fallowfield, Jenkins, and Beveridge 2002), or more generally the use of placebo treatments in medical practice (Chen and Johnson, 2009; Hull et al. 2013). Similar studies have also been carried out on physician attitudes toward deception in these contexts (Howick et al. 2013; Lynöe, Mattsson, and Sandlund 1993).

However, several important dimensions of deception in medicine have not yet been addressed. Previous empirical studies have not directly compared patient attitudes toward deception across different medical contexts, nor have they investigated the relationship between patient attitudes toward deception in medicine and their attitudes toward truthfulness in nonmedical contexts. It remains unclear whether observed attitudes to deception reflect more general views about deception or whether they are specific to the medical sphere or even to particular medical contexts.

In addition, and perhaps most critically, previous studies have not investigated patient attitudes toward different methods of deception in specific medical contexts. This is an important lacuna in the literature, especially in view of the long-standing debate in medical ethics concerning whether some methods of deception are worse than others. For example, some ethicists have argued that it is worse to lie than to deceive someone by choosing not to tell that person information in order to get that person to form a false belief (Benn 2001; Gillon 1993; Jackson 1991); other ethicists argue that the two are morally equivalent (Bakhurst 1992).

One difficulty that arises in addressing these questions is that individuals are rarely exposed to a wide range of medical contexts; as such, it is not possible to directly examine patient attitudes toward deception across different medical contexts. In view of this unavoidable limitation, the aim of this study was to investigate the attitudes of lay people toward the moral acceptability of deception across different medical contexts. We examined lay attitudes toward the moral acceptability of different methods of deception, the relationship between their attitudes toward the moral acceptability of different sorts of deception across different medical contexts, and their attitudes toward the moral acceptability of deception in nonmedical contexts. In doing so, we hope to provide more fine-grained information about when and how laypersons believe it is acceptable to be deceived in medical contexts.

## Methods

### Participants

Respondents to the survey were recruited by posting the survey on the Amazon “Mechanical Turk” website (www.mturk.com). This website is an online crowd-sourcing service that advertises tasks that users can complete in return for compensation. Studies have suggested that this web-site can be used to obtain high-quality, reliable data (Buhrmester, Kwang, and Gosling 2011; Gosling et al. 2004). The survey was only made accessible to U.S. citizens who had earned a Mechanical Turk “master qualification.” According to the website, this qualification is awarded to:

elite groups of Workers who have demonstrated accuracy on specific types of [tasks] on the Mechanical Turk marketplace … by consistently completing [tasks] of a certain type with a high degree of accuracy across a variety of Requesters. (Amazon Mechanical Turk FAQs 2015)

To screen out participants who had not paid sufficient attention to the survey, we included a dummy question that we asked respondents not to answer. The attention-check question we used is based on standard validated practice (Oppenheimer, Meyvis, and Davidenko 2009) and is regularly used in Mechanical Turk studies. Those who completed the survey received a small financial award of $0.50. The study was approved by the Social Sciences and Humanities Inter-Divisional Research Ethics Committee at the University of Oxford. All participants signed an online consent form.

### Survey instrument

In order to investigate the attitudes of laypersons toward different sorts of deception in medical care, respondents were presented with a series of vignettes, each followed by several questions. The questionnaire we developed asked respondents to indicate on a Likert scale their attitudes toward the behavior of physicians in various vignettes involving some form of deception (with a response of 1 representing completely unacceptable and 7 representing completely acceptable). In our descriptive analysis, we then divided the scale into three groups: Responses of 1–3 indicated a belief that the act in question is unacceptable and responses of 5–7 indicated a belief that the act in question is acceptable. A response of 4 was taken to indicate that the respondent was ambivalent toward the acceptability of the act in question. The second half of the questionnaire asked respondents to answer questions about their general moral attitudes.

We received feedback on the vignettes in the first half of the survey prior to implementation from a clinical geneticist, an orthopedic surgeon, a neurologist and two GPs—all with considerable experience relating to the dilemmas presented in the survey. As a result of this consultation, we made a change to the condition involved in one of the vignettes in order for the vignette to more accurately reflect clinical reality.[Fn fn0001] ^1^Editor's note: The full survey is available online as Supplemental Material.


It should be acknowledged that we asked participants only about the acceptability of certain courses of action, and not about what doctors should do or about what course of action participants most prefer. This is important, since even if the majority of laypersons think that some level of disclosure is the most acceptable course of action in a specific context, this should not be understood to entail that they also think that providing this level of disclosure would be the morally best course of action, or what the participants most prefer. Further research would be required to establish that this is the case.

### Placebo questions

The first vignettes concerned the use of deceptive placebos in clinical care. In two of the three placebo vignettes—“Cold” and “Neck”—a doctor was presented as believing that she could use a sugar pill, that is, a pure placebo, to elicit the placebo effect and that this is the only available way to alleviate the patient's current suffering. However, “Cold” and “Neck” differed with respect to the nature of the ailment for which the placebo was being prescribed. In “Cold,” the patient was suffering from a particularly bad cold that the physician believed would go away in a few days. In “Neck,” the patient was complaining of chronic neck pain. Following each vignette, we asked respondents to give their opinions on the acceptability of different courses of action that the physician could take in these situations.

It should also be acknowledged that in the first two vignettes, we stipulated that the physician believed that the healing effect of the placebo would be strongest if the patient believed that the treatment that he was receiving was an active therapy. We also pointed out that the physician believed that the patient's condition could not be cured using an active therapy. Finally, we included a basic description of how the placebo effect would work, explaining that the doctor was aware of research suggesting that the placebo therapy would make the patient in the vignette feel better not because of any active ingredients in the substance, but rather because of the patient's belief that taking the substance would help.

The first course of action that we asked respondents about in each of these two vignettes involved the physician prescribing a placebo after providing a partial disclosure the nature of the treatment to the patient. Here, the physician gave the patient a sugar pill and told them, truthfully, “I believe that this pill will help you feel better because it has helped other people in your condition feel better,” but they did not tell the patient that the pill is a sugar pill and will not help to fight what is causing the patient's symptoms (“partial disclosure”). It should be noted that in their 2013 study, Howick and colleagues found that around half GPs who had prescribed a placebo at some point in their careers (97% of respondents) had done so having given their patients such partial disclosure.

The second course of action that we asked respondents about involved the physician prescribing the placebo and explicitly lying to the patient by telling the patient that the pill the patient was receiving was an active therapy that would help to fight what was causing the patient's symptoms (“lying”). The third and final course of action that we asked respondents about involved the physician's deciding to not prescribe the placebo, and instead telling the patient that there are no available active medications for the patient's condition (“nonprescription”).[Fn fn0002] ^2^It should be noted that in their study of patient attitudes toward physician and patient attitudes toward placebo use, Fassler and colleagues (2011) included questions about courses of action similar to “partial disclosure” and “lying” but not “nonprescription.”


In a third vignette, “Skin,” the patient was suffering from a skin condition that the doctor believed was a result of anxiety. The doctor had to choose between treating the patient with a steroid cream that has negative side effects or a placebo moisturizer that contains no active medical ingredients. The doctor was also described as believing that the symptoms will probably soon stop naturally in a few days anyway. This question was designed to elicit laypersons' attitudes toward non-pill-based pure placebos when an active therapy with negative side effects is also available.

### Sham surgery questions

The fourth vignette focused on patients in a clinical trial that they believe will involve undergoing an arthroscopy. However, to investigate the extent to which the placebo effect might contribute to the therapeutic efficacy of arthroscopies, researchers ensure that one group of patients will undergo a procedure that is identical to an arthroscopy, but that crucially does not involve the part of the procedure that is understood to be therapeutically efficacious, namely, the removal of bone fragment. Respondents were first asked whether it would be acceptable to allow patients to participate in such a trial. They were then asked whether it would be acceptable to deceive patients in the trial if doing so would allow researchers to better answer the question of whether the removal of the bone fragment is necessary for the surgery to be therapeutically efficacious.

### Therapeutic privilege questions

We asked respondents to give their opinions about physicians invoking the so-called therapeutic privilege, which allows them to withhold medical information from patients if they believe that disclosures will cause undue distress to these patients. In the first vignette, “Cancer,” an 85-year-old woman is diagnosed with terminal cancer; the family asks the doctor not to inform her of this diagnosis and the fact that she will die in a few weeks, as they believe that she will not be able to cope with this information. In the second vignette, “Motor Neuron Disease,” a doctor diagnoses a man with motor neuron disease; there is no family involvement in the doctor's decision about what to disclose. Again, we asked respondents to give their opinions about the acceptability of different courses of action that the physician could take.

Although there were two differences between these two vignettes (family involvement and type of condition), we attempted to control for the difference between the types of condition in the vignettes by using conditions that would both have devastating consequences. In the first vignette, we claimed that the woman had terminal cancer and would die in a couple of weeks; in the second, we explained that motor neuron disease is a fatal disease that would make the patient become weaker and weaker over a period of a year to a few years, eventually leading to him being unable to move or swallow. We pointed out that eventually he will be unable to breathe and will die unless placed on a breathing machine.

### General attitudes to truth-telling questions

In addition to the vignette-based questions, we asked respondents whether it is worse to make someone believe something that is not true by providing them with false information or by giving them true information that is likely to lead them to develop a false belief while omitting other important information that shows the belief to be false. Respondents had the option of claiming that the two methods were morally equivalent.

## Results

Two hundred respondents completed the questionnaire; all of them passed the attention check. The age range of respondents was 18 years and above, with the majority of respondents aged 25–34. A majority (64.5%) of the respondents were female.

### Placebo questions

The results of questions pertaining to the placebo vignettes (Table 1) are striking in a number of ways. Although the “nonprescription” option received the greatest support of the available options, the majority of our respondents found partial disclosure to be acceptable in “Cold” and “Neck” (50.5% and 55%, respectively). Strikingly, our results also suggest that the majority of respondents reject outright lying. In “Cold,” 82.5% claimed that the course of action we call “lying” would be unacceptable; in “Neck,” 75.5% claimed that it would be unacceptable.

**Table 1.** Results for placebo vignettes.

		Percentage (%) of responses by vignette		
		Cold	Neck	Skin
Partial disclosure	Acceptable	50.5	55	80.5
	Ambivalent	11	12.5	10.5
	Unacceptable	38.5	32.5	9
Lying	Acceptable	11.5	13	8
	Ambivalent	6	11.5	10.5
	Unacceptable	82.5	75.5	81.5
Nonprescription	Acceptable	91	82.5	N/A
	Ambivalent	5	10	N/A
	Unacceptable	3.5	7.5	N/A
Prescribe active treatment	Acceptable	N/A	N/A	40
	Ambivalent	N/A	N/A	23
	Unacceptable	N/A	N/A	37
Prescribe inert treatment	Acceptable	N/A	N/A	78
	Ambivalent	N/A	N/A	12.5
	Unacceptable	N/A	N/A	9.5

Our results here are in keeping with other comparable studies. For instance, Fassler and colleagues (2011) found that patients believe that prescribing a pure placebo on the basis of a direct lie is worse than prescribing one on the basis of indirect information (50% objected to the former, while only 29% objected to the latter). Similarly, Hull and colleagues (2013) used a vignette involving a physician giving a partial disclosure before prescribing a placebo and found that 36.6% of their respondents thought that this was definitely acceptable, 29% thought it probably was, 9.7% thought that it probably was not, and 24.7% thought it definitely was not.

There were some interesting differences between the results of the related questions across “Cold” and “Neck.” For instance, the difference between the mean responses for the questions pertaining to partial disclosure in “Cold” and “Neck” was statistically significant (*t* = 3.68(199), *p* < .000), with respondents finding partial disclosure more acceptable in “Neck” than in “Cold.” This suggests that laypersons may be more supportive of placebo use for chronic pain than discomfort due to self-limiting illness. However, the difference between the mean responses for the questions pertaining to [outright] lying in “Cold” and “Neck” was not statistically significant. It could be hypothesized that where the condition is more serious or persistent, such that the patient has more to gain from alleviation of his or her symptoms, respondents were more willing to accept some partial deception, although this did not extend to greater acceptance of outright lying.

Similarly to “Cold” and “Neck,” the results in “Skin” suggest that the majority of respondents believed that placebo use was acceptable if it involved no deception (either by lying or partial disclosure). In contrast, though, respondents found it less acceptable to prescribe the steroid cream, perhaps because patients had not been offered the alternative of a benign moisturizer that they believe would not lead to adverse side effects. It appears that laypersons are divided with regard to the acceptability of physicians prescribing an active medication that has negative side effects when the physician believes that he or she could prescribe an inert placebo that is likely to achieve the same results.

Our results strongly suggest that:

1. Lay people are strongly opposed to outright lying.
2. The majority support partial disclosure when it may benefit the patient.
3. There is strong support for physicians fully disclosing the fact that no active treatment exists for a particular condition.

### Sham surgery

The strong possibility that the placebo effect can be elicited by surgical procedures (Beecher 1961; Ernst and Resch 1995; Freed et al. 2001; Freeman et al. 1999; Johnson 1994; Wolf and Buckwalter 2006) raises further ethical questions. First, there is a substantially greater risk of harm involved in undergoing a surgical procedure than there is from taking a pure placebo; while a sham surgical procedure carries risks of infection or complications, a pure placebo is an inert substance.[Fn fn0003] ^3^It should also be acknowledged that a surgical procedure that is not intended to be a sham surgery but is rather incorrectly believed to be effective also carries significant risks.


Second, one of the most salient moral issues that the surgical placebo effect raises pertains to the context of medical research. In order to investigate the efficacy of a new drug, researchers carry out a double-blind randomized placebo-controlled trial, where the effect that taking the drug has on one group of subjects is compared to the effect that taking a placebo has on a control group of subjects. The idea underlying this research method is that by comparing the results of the two groups, researchers can estimate how much the new drug's effect can be accounted for by the placebo effect. However, very few surgical procedures are subjected to this form of investigation (Angelos 2003; Miller 2003; Wolf and Buckwalter 2006).

In view of this, and in view of the evidence suggesting that surgical procedures can elicit the placebo effect, it is possible that researchers could conduct trials of new surgical techniques in which a control group receive a placebo surgical intervention, or “sham surgery,” in which the surgeon carries out the procedure under investigation but omits the steps that are thought to be therapeutically necessary.

The use of sham surgery in the research context has provided results that suggest that the therapeutic efficacy of a number of procedures is due to the placebo effect. For instance, Kim and colleagues (2005) used sham-controlled experiments to show that the apparent efficacy of cell-transplant surgical interventions in the treatment of Parkinson's disease was due to the placebo effect. Moreover, recent research also suggests that arthroscopic meniscal tear surgery is no better than sham surgery (Sihvonen et al. 2013).

In spite of these findings, many have raised ethical objections to the use of sham surgery in clinical trials, since it seems to violate the physician's duty of nonmaleficence. We believe these objections are misplaced because performing ineffective surgery, in the belief that it is effective, also exposes patients to risks without benefits. For this reason, trials are necessary (Wartolowska et al. 2014). Given that very large numbers of patients can be exposed to risky surgical innovations that have not been evaluated by clinical trials, the potential harm of “business as usual” is significant (Miller 2003). However, in view of these ethical considerations regarding sham surgery, and the fact that very few empirical studies have been carried out on lay attitudes to sham surgery (Swift 2012), we included a question asking respondents about the acceptability of using sham surgery in a clinical trial.

Our findings (Table 2) suggest that respondents tended to support such research when participants had agreed to it. This goes against the professional ethical tide of suggesting that such research is problematic. Furthermore, although the majority of respondents rejected deceiving participants in a research context, a surprisingly high percentage (35.5%) believed that it would be acceptable. This is especially surprising in comparison to respondent attitudes toward deceptive placebo use in a clinical setting. Analysis of the responses to the questions pertaining to “Research” and the responses to the questions pertaining to “Cold,” “Neck,” and “Skin” suggests that respondents regarded the use of deceptive placebos in the context of clinical research as more acceptable than their use in a clinical practice context, with paired-samples *t*-tests showing these differences to be statistically significant ([“Research” cf. “Cold”] *t*(199) = 7.877, *p* < .001; ([“Research” cf. “Neck”] *t*(199) = 7.409, *p* < .001; ([“Research” cf. “Skin”] *t*(199) = 8.467, *p* < .001).

**Table 2.** Sham surgery results.

		Percentage (%) of responses
Acceptable to allow participation	Acceptable	55.5
	Ambivalent	13.5
	Unacceptable	31
Acceptable to deceive participants	Acceptable	35.5
	Ambivalent	9.5
	Unacceptable	55.5

### Therapeutic privilege

The data (Table 3) suggest that respondents were somewhat divided over the acceptability of partial disclosure in the interests of the patients, but tended to be supportive of it; however, they were again clearly against lying. Interestingly, respondents were more supportive of fully involving the patient (and not deferring to family) in the case of the more chronic motor neuron disease. It is plausible that because of the longer time course of the disease in the “Motor Neuron Disease” example, respondents were more supportive of actively informing because the information would be of greater utility to the patient, and hence more desired, in terms of planning time, organizing affairs, and living as well as possible.

**Table 3.** Therapeutic privilege results.

		Percentage (%) of responses by vignette	
		Cancer	Motor Neuron
Full truth	Acceptable	47.5	95.5
	Ambivalent	15.5	3.5
	Unacceptable	37	1
Basic truth, with for questions	Acceptable	70	50
	Ambivalent	11.5	13
	Unacceptable	18.5	37
Say nothing (lie in Cancer)	Acceptable	7	3
	Ambivalent	9	5
	Unacceptable	84	92
Truth with positive outlook	Acceptable	47.5	N/A
	Ambivalent	19	N/A
	Unacceptable	33.5	N/A
Say nothing unless asks	Acceptable	33.5	N/A
	Ambivalent	20	N/A
	Unacceptable	46.5	N/A
Ask family what to tell	Acceptable	N/A	21.5
	Ambivalent	N/A	11
	Unacceptable	N/A	67.5

Our results here are congruous with comparable studies. For instance, Fallowfield and colleagues (2002) found that the terminally ill prefer to have information about their conditions. Furthermore, Benson and Britten (1996) examined the attitudes of UK cancer patients, finding that such patients rejected unconditionally their family influencing what information they would be given.

### General attitudes to truth-telling

These responses (Table 4) point to a possible discrepancy among our respondents' general attitudes toward truthfulness. The data suggest that our respondents were divided on the abstract theoretical question of whether it is worse to deceive someone by lying to them than by omitting important information, or whether the two types of deception are morally equivalent. However, our other findings strongly suggest that the majority of our respondents did not believe that the two methods of deception were morally equivalent when considering their application in specific medical contexts; while many of our respondents regarded deception through partial disclosure to be acceptable in some cases, very few were prepared to support doctors outright lying to their patients.

**Table 4.** Highlight which of the following you think is worse.

	Percentage (%) of responses
(a) Providing patients with false information	41.5
(b) Giving patients true information that is likely to lead them to form the false belief, without telling them other important information that shows it to be false.	16
(c) Are (a) and (b) morally equivalent?	42.5

## Discussion

We found that respondents most widely supported nonprescription of placebos, a majority supported placebo use with partial disclosure, and the vast majority of our respondents were against outright lying. Respondents were more supportive of placebo use for chronic pain than for discomfort due to self-limiting illness. Our results also suggest that where there are competing alternatives with different risk–benefit profiles, patients believe that they ought to be informed of these options.

Strikingly, a majority supported sham surgery as a research tool. Although the majority of respondents believed that it would be unacceptable to deceive participants in such a trial, a considerable percentage believed that it would be acceptable. Indeed, our respondents were more accepting of deception in research than they were of deception in clinical practice to a statistically significant degree.

With respect to therapeutic privilege, respondents supported fully informing patients, especially in chronic conditions. There was some support for partial disclosure, though a clear majority of our respondents were against lying. Interestingly, respondents' general moral attitudes toward truthfulness in a nonmedical context did not necessarily map onto their attitudes toward deception in medicine.

Our primary aim in this study was to find out what proportion of laypersons believe that deception is acceptable in different medical contexts. There are obviously limits to how far we can extrapolate from these results. Our questions asked respondents to make a judgment about when deception is morally acceptable. These responses do not necessarily entail that our respondents would always want to be deceived in similar circumstances. For instance, it may be easier to approve the deception of another person than the deception of oneself. Nor do these responses necessarily indicate that our respondents thought that deception is necessarily the morally best course of action in the situations described.

Our study does, however, cast doubt over endorsing a deontological principle that absolutely prohibits deception in medical ethics, such as that which is seemingly endorsed by the AMA. While the data suggest that our respondents believed that deception was acceptable in some circumstances, the differences in their responses to different sorts of deception across the various vignettes suggest that they appear to consider a range of values in their assessment of the permissibility of deception in medicine, especially when compared to professional ethical discussions, which often claim that deception is unethical in all circumstances. We therefore suggest that it would be advisable to reconsider the commonly accepted deontological view in medical ethics that deception is always morally impermissible. If medical norms ought to at least partly reflect the views of laypersons, the assumption that deception is always unacceptable is directly challenged by our findings.

Furthermore, our data also suggest that our respondents accept an important moral distinction between different methods of deception. Whilst the majority of our respondents were opposed to lying (except, to some degree, for research purposes), they were prepared to support partial disclosure and the use of placebos when it is in patients' interests.

One interesting theoretical point that this finding raises is that while our respondents seemed to adopt a broadly consequentialist attitude toward the acceptability of deception, they also believed that the manner in which agents are deceived is relevant to the moral assessment of deception. This is interesting because consequentialists would tend to deny the latter claim; from a consequentialist perspective, all that matters with regard to the moral acceptability of deception is whether or not the deception brings about good consequences.

At a more practical level, we believe that this data calls for more nuanced future discussions of the moral acceptability of deception in medicine; future discussion should acknowledge the importance of distinguishing the moral acceptability of different methods of deception.

### Limitations

It should be acknowledged that there are several important limitations to the present study. First, our sample size of 200 was relatively small and included only U.S. citizens. This is an important limitation, since attitudes toward deception are likely to vary across different cultures.

Second, the study assessed lay attitudes to imagined scenarios rather than patient attitudes to real-life scenarios. As we have already explained, since our survey covered a broad range of medical contexts, it was not possible for us to directly examine the views of patients who suffer from these medical conditions, and the data we report instead reflect the views of our sample of laypersons. It cannot be ruled out that patients with these (or other) specific conditions would have somewhat different views about being deceived.

Third, a related limitation is that, for greater realism, we presented the vignettes in the third person. It is at least possible that respondents' attitudes to deception would have been somewhat different if they directly considered that they themselves would be deceived. Finally, since the order of the vignettes and questions was not randomized, it is possible that the answers to some questions were influenced by answers to previous ones. There is room for future research that will investigate whether responses change if questions are presented in the first person and in a different order.

In spite of these limitations, we believe that this study represents an important contribution to the empirical literature on the acceptability of deception in medicine because it investigates both lay attitudes to deception across different medical contexts and lay attitudes to different methods of deception in medicine. These issues have not received adequate attention in the existing literature.

## Conclusion

We have provided some population level data for laypersons' attitudes toward deception in a range of medical contexts. As we pointed out in the introduction, debates concerning the moral acceptability of deception in medicine can be understood to represent a conflict between the principle of respect for autonomy and the principle of beneficence. Since our data show that the majority of our respondents endorse some forms of deception, our data might be understood to suggest that our respondents believed that the principle of beneficence ought to win out in this conflict.

However, an interesting alternative interpretation of the data is that our respondents did not believe that the scenarios that we considered involved conflicts between the principles of autonomy and beneficence. While we did not ask respondents whether they personally would want to be deceived in the scenarios we presented, it seems plausible to hypothesize that there might be some relation between a judgment that deception is morally acceptable in some scenario, and what an agent would prefer if that person were in that scenario.

Moreover, although holding that deception is morally acceptable in this context is compatible with nevertheless overall preferring some other course of action to be taken, we do believe that the fact that the majority of our participants regarded some forms of deception to be acceptable is strong evidence that these participants do not strongly want to be told the full truth in all relevant medical situations. They may, for example, prefer to leave it to the discretion of the doctor whether deception should be used or not, as long as it is in their interests.

Our data suggest that it is doubtful that deception in such cases would be strongly opposed to these participants' wishes. This, we believe, may be sufficient for autonomy to be respected in these cases. Our results thus further suggest (though less conclusively) that the views of many laypersons also support an autonomy-based justification for certain deceptive practices in medical contexts, as well as a justification based on the principle of beneficence. Further research is needed to establish whether such an autonomy-based justification of deception in medicine could be plausible.

In conclusion, if future research shows that patients would also want to be deceived in a wide range of medical contexts, then this, in conjunction with the data presented here, suggests that we should allow physicians a greater degree of ethical latitude and authority to make a professional judgment about whether deception is morally warranted by the circumstances, as long as it does not involve outright lying. These judgments would need to be guided both by an assessment of whether deception is in the interests of the parties concerned and whether the patient herself would want to be deceived.

While we have not made the full case for this position here, we believe that we have at least begun to challenge the prevailing deontological orthodoxy against deception in medical ethics. There is no single policy that will suit all patients in all circumstances, as our respondents realized; rather, we believe that future research should aim to establish a practicable policy that permits doctors to engage in a range of practices using placebos and partial disclosure.

Such a policy could be both ethical and acceptable to patients if tailored for patient-specific data and governed by assessment of whether deception is in the interests of the parties concerned and whether patients would want to be deceived in the scenario in question. Importantly, however, precautions would need to be put in place to guard against the use of deception in ways that violate these constraints (e.g., because deception is convenient for the doctor, rather than because it would genuinely benefit the patient).

## Supplementary Material

Survey Online Supplement
