# Supplementary material for: Lay attitudes toward deception in medicine: Theoretical considerations and empirical evidence
Source: AJOB Empir Bioeth. 2015 Nov 16;7(1):31–8. doi: 10.1080/23294515.2015.1021494 (PMC4673517; doi:10.1080/23294515.2015.1021494)
Supplement: Survey Online Supplement [file uabr_a_1021494_sm3187.pdf]

Truth Telling in Medicine

Thank you for considering participating in this study.

This research project is conducted by the Uehiro Centre for Practical Ethics, Oxford. The principle investigators are Dr. Guy Kahane and Professor Julian Savulescu. The researcher conducting this research is Jonathan Pugh: (jonathan.pugh@philosophy.ox.ac.uk). This research will eventually be written up as a research paper for scholarly publication.

This study will consist of a simple electronic questionnaire, to be completed online. We are investigating how people think about deception in medical care. Completing this study should take approximately 20 minutes. If, for any reason, you prefer not to complete the study, we respect your choice to terminate your involvement. However, if you choose to complete the questionnaire your cooperation is greatly appreciated.

Mechanical Turk Payment: 0.50 USD

Please note that this HIT may be periodically re-posted. If you've already completed this HIT previously, please do not complete it a second time. You will not be compensated a second time.

All the data will be stored in conformity with the Data Protection Act and will be anonymized after completion of the study. If the paper we develop from this research is accepted for publication, it will be published in both print and online form. Depending on the journal, it may be published with open access and available to every Internet user.

This project has been reviewed by, and received ethics clearance through the University of Oxford Central University Research Ethics Committee. If you have a concern about any aspect of this project, please speak to the researcher Jonathan Pugh who will do his/her best to answer your query. If you remain unhappy or wish to make a formal complaint, please contact the Research Ethics Committee at the University of Oxford ethics@socsci.ox.ac.uk

Please not that you may only partcipate in this survey if you are 18 years of age.

If you are happy to proceed, then please confirm the following:

***1. I have read and fully understood all of the information given to me on this screen**

◯ Yes

◯ No

***2. I can confirm that I am 18 years of age or over**

◯ Yes

◯ No

Please read the scenario, indicate your response to each question by highlighting a number on the scale.

• Imagine that you feel bad like you have the flu. Suppose that your doctor believes that you have a particularly bad cold which will go away in a few days and which cannot be cured with drugs. However, she is aware of research that has shown that that she can make you feel better by giving you a sugar-pill. This pill looks like a normal drug, but it is made of sugar and does not contain any medically active ingredients (it also contains very few calories). The sugar pill is likely to make you feel better, not because of anything in the pill, but rather because you believe that taking a pill will help. The doctor believes that the healing effect of the sugar pill would be strongest if you believe that the pill she is giving you is a real drug (and not a sugar pill) containing active ingredients which would fight the virus causing your symptoms.

To what it extent would it be permissible for the physician to act in each of the following ways:

***3. Give you the sugar pill and say to you, truthfully, "I believe that this pill will help you feel better because it has helped other people in your condition feel better", but omit to tell you that the pill is a sugar pill and will not help to fight the virus which is causing your symptoms?**

1 - Completely Unacceptable	2	3	4	5	6	7 - Absolutely Acceptable
○	○	○	○	○	○	○

***4. b) Give you the sugar pill and say to you, falsely, "I am giving you a drug that will fight the virus that is causing your symptoms"?**

1 - Completely Unacceptable	2	3	4	5	6	7 - Absolutely Acceptable
○	○	○	○	○	○	○

***5. Not give you the sugar pill, and tell you, truthfully, "Unfortunately, there are no drugs that can fight the virus that is causing your symptoms, but you should get better in few days time"?**

1 - Completely Unacceptable	2	3	4	5	6	7 - Absolutely Acceptable
○	○	○	○	○	○	○

• A married couple bring their son to doctors with an abnormality. During their investigations, the doctors find that the child could not be the son of the man (the husband of the child's mother). He believes he is the genetic father.

***6. In your view, how ethically permissible would it be for the doctor to choose to do each of the following:**

	1 - Completely Unacceptable	2	3	4	5	6	7 - Absolutely Acceptable
a) Discuss this confidentially with the mother?	○	○	○	○	○	○	○
b) Ignore it on the basis that it is not relevant to the child's abnormality?	○	○	○	○	○	○	○
c) Encourage the mother to reveal this finding to her husband and explain it?	○	○	○	○	○	○	○
d) Inform the husband that he is not the father of the child, even if the woman does not want them to?	○	○	○	○	○	○	○

• Patients often complain of shoulder pain. If pain persists despite physiotherapy and injections, then surgeons can perform an “arthroscopy” (inserting a flexible telescope into the shoulder) and remove small bone fragments in an attempt to relieve pain. The surgery appears effective at relieving pain. However, some doctors believe this is not due to the removal of the bone fragments, but due the attention the patient receives, the rest and the physiotherapy they receive afterwards. They argue that patients might be being exposed to unnecessary risks of surgery and anaesthesia. They propose to settle whether the surgery works or if it is related to other factors. They propose a clinical trial where patients are divided into 2 groups. Both will receive all parts of the care plan but half will have the arthroscopy without removal of the bone fragment. That is, they will have an incision and an arthroscopy, but nothing will be done unless a more specific abnormality is seen. If a person agrees to take part, a coin will be tossed to decide which treatment the patient will receive.

***7. In your view, to what extent do you believe that it would be acceptable for patients be allowed to participate in such a trial?**

1 - Completely Unacceptable	2	3	4	5	6	7 - Absolutely Acceptable
○	○	○	○	○	○	○

***8. Imagine that the above trial will be better able to answer the question of whether removal of the bone fragment is necessary if patients believe that they are receiving full surgery, including removal of bone fragments, even if in fact they are not receiving any surgery. To what extent would it be acceptable for doctors to be allowed to deceive patients in the trial so that they believe they will be getting the full surgical procedure when they will not be getting it?**

1 - Completely Unacceptable	2	3	4	5	6	7 - Absolutely Acceptable
○	○	○	○	○	○	○

• An 85 year old woman is diagnosed with terminal cancer. Her family says to doctors that she would not want to know that she has cancer. They ask doctors not to reveal her diagnosis and the fact that she will die in a matter of weeks. They say she would not be able to cope with knowing this information.

*9. In your view, how ethically acceptable would it be for the doctor to:

	1 - Completely Unacceptable	2	3	4	5	6	7 - Absolutely Acceptable
a) Tell her the diagnosis anyway and tell her clearly she will likely die in a matter of weeks?	◯	◯	◯	◯	◯	◯	◯
b) Tell her she has a serious illness but let her ask questions about it, answering these honestly?	◯	◯	◯	◯	◯	◯	◯
c) Tell her she has cancer and answer her questions with a positive outlook?	◯	◯	◯	◯	◯	◯	◯
d) Not tell her anything unless she asks, but in that case tell her the truth?	◯	◯	◯	◯	◯	◯	◯
e) Not tell her anything and lie if she asks whether she has cancer or is likely to die?	◯	◯	◯	◯	◯	◯	◯

Imagine that you have experienced considerable pain in your neck for a few years. After examining you, your doctor understands what is physically wrong with your neck, but knows that she cannot do anything to change it. However, she believes that she can get rid of the pain by getting you to take sugar pills once a week for the next few months. These pills look like normal drugs, but they are actually made of sugar and do not contain any medically active ingredients. However, the doctor is aware of research which suggests that if you take the sugar-pill believing that it is a real medically active drug, then the sugar pill can help take away the pain caused by the problem in your neck. The doctor believes that the healing effect of the sugar pill would be strongest if you believe that the pill she is giving you is a real drug containing active ingredients.

To what it extent would it be acceptable for the doctor to act in the following ways:

***10. Give you the sugar pill and say to you, truthfully, “I believe that this pill will help you feel better because it has helped other people in your condition feel better”, but omit to tell you that the pill is a sugar pill and will not help to fix what is physically wrong with your neck?,**

1 - Completely Unacceptable	2	3	4	5	6	7 - Absolutely Acceptable
○	○	○	○	○	○	○

***11. Give you the sugar pill and say to you, falsely, “I am giving you a drug that will fix the problem in your neck that is causing your symptoms”?**

1 - Completely Unacceptable	2	3	4	5	6	7 - Absolutely Acceptable
○	○	○	○	○	○	○

***12. Not give you the sugar pill, and tell you, truthfully, “Unfortunately, there are no drugs that can fix the problem with your neck that is causing your symptoms; you will have to learn to live with it”?**

1 - Completely Unacceptable	2	3	4	5	6	7 - Absolutely Acceptable
○	○	○	○	○	○	○

• A married couple bring their son to doctors with an abnormality. During their investigations, the doctors find that the child could not be the son of the man (the husband of the child's mother). He believes he is the genetic father.Suppose that the man asked doctors whether they could tell whether the boy is his son.

***13. In your view, how ethically acceptable would it be for the doctor to do only the following :**

	1 - Completely Unacceptable	2	3	4	5	6	7 - Absolutely Acceptable
a) Ask permission to reveal the truth from the mother?	◯	◯	◯	◯	◯	◯	◯
b) Tell the truth, regardless of the mother's wishes?	◯	◯	◯	◯	◯	◯	◯
c) Lie, and say that the boy is the man's son?	◯	◯	◯	◯	◯	◯	◯
d) Lie, and say they can't tell whether or not the boy is the man's son?	◯	◯	◯	◯	◯	◯	◯
e) Tell the truth only if the mother lets them?	◯	◯	◯	◯	◯	◯	◯
f) Do what the doctors judges to be in the best interests of the boy.	◯	◯	◯	◯	◯	◯	◯

A 50 year old man develops increasing weakness. His doctor performs tests and realises that the patient has a terrible fatal disease - motor neurone disease (Lou Gehrig's disease). He will become weaker and weaker over a period of a year to a few years, and will eventually be unable to move or swallow. He will then need to be fed by a tube. Finally he will be unable to breathe and will die unless placed on a breathing machine.

***14. In your view, how ethically acceptable would it be for the doctor to do the following:**

	1 - Completely Unacceptable	2	3	4	5	6	7 - Absolutely Acceptable
a) Tell him the diagnosis and what will happen to him?	◯	◯	◯	◯	◯	◯	◯
b) Tell him that he has a condition that will cause increasing weakness and wait for him to ask questions?	◯	◯	◯	◯	◯	◯	◯
c) Ask his family what he should be told?	◯	◯	◯	◯	◯	◯	◯
d) Say nothing but ask to review him in 6 months, saying they will see how it goes?	◯	◯	◯	◯	◯	◯	◯

Imagine you go to a doctor with a sore throat which is causing you a great deal of pain. It is late in the afternoon, and the doctor wants to go home. After asking you a couple of questions, the doctor gives you a packet of sugar pills and tells you that they should cure your symptoms. He does not believe that the pills will significantly help you, but he knows that they will not harm you; it will also allow him to finish the consultation early.

***15. In your view, how ethically acceptable would it be for the doctor to tell you this and give you a sugar pill?**

1 - Completely Unacceptable	2	3	4	5	6	7 - Absolutely Acceptable
○	○	○	○	○	○	○

Studies have shown that up to 20% of children are not the child of the man in the family who believes he is the genetic father. In addition, in some rare cases “baby mix ups” can occur in hospital or during IVF.

***16. Do you think that all children should be given paternity testing soon after birth and the “father” informed of the result?**

Strongly Disagree	Disagree	Slightly Disagree	Slightly Agree	Agree	Strongly Agree
◯	◯	◯	◯	◯	◯

Issues of truthfulness are important in medicine. This question is important to verify that participants are truthfully responding.

17. To confirm that you are reading this text, please do not select any of the option in the scale below.

1 - Completely Unacceptable	2	3	4	5	6	7 - Absolutely Acceptable
◯	◯	◯	◯	◯	◯	◯

A patient presents with a skin condition that the doctor believes is the result of anxiety. The doctor could prescribe a steroid cream, which has negative side effects, or a moisturizer that contains no medically active ingredients. If the doctor prescribes the moisturizer, the doctor believes that the patient will not experience any negative side effects, and that the skin condition will probably soon stop naturally.

In your view, how ethically acceptable would it be for the doctor to:

***18. Prescribe the moisturizer, tell the patient that she is being prescribed moisturizer, and see if the condition goes away.**

1 - Completely Unacceptable	2	3	4	5	6	7 - Absolutely Acceptable
○	○	○	○	○	○	○

***19. Prescribe the moisturizer tell the patient, truthfully, that other patients with a similar condition have found that their skin condition goes away after they have used the moisturizer, and see if the condition goes away.**

1 - Completely Unacceptable	2	3	4	5	6	7 - Absolutely Acceptable
○	○	○	○	○	○	○

***20. Prescribe the moisturizer and tell the patient she is being prescribed steroids and see if the skin condition goes away.**

1 - Completely Unacceptable	2	3	4	5	6	7 - Absolutely Acceptable
○	○	○	○	○	○	○

***21. Just prescribe the steroids**

1 - Completely Unacceptable	2	3	4	5	6	7 - Absolutely Acceptable
○	○	○	○	○	○	○

General Moral Attitudes

Pages 13-16 of this survey contain questions concerning your general moral attitudes

Suppose that the husband of your close friend Jane told you that he had a secret romantic involvement with another woman four years ago but would never want to do so again, since his marriage means so much to him. On a night out with Jane she mentions that she was worried about her marriage four years ago. Jane goes on to say that her marriage is immensely important to her and that if her husband was ever unfaithful it would destroy the marriage because she would never be able to trust him again. She asks whether you've ever heard anything to suggest her husband isn't faithful.

*** 22. Should you tell Jane that her husband had an affair?**

1 - Deeply wrong	2	3 - Somewhat wrong	4	5 - Not wrong at all
◯	◯	◯	◯	◯

Imagine a runaway trolley is heading down the tracks toward five workmen who will be killed if the trolley proceeds on its present course. You are on a footbridge over the tracks, in between the approaching trolley and the five workmen. Next to you on this footbridge is a stranger who happens to be very large. The only way to save the lives of the five workmen is to push this stranger off the bridge and onto the tracks below where his large body will stop the trolley. The stranger will die if you do this, but the five workmen will be saved.

***23. Should you push the stranger on to the tracks in order to save the five workmen?**

1 - Deeply wrong	2	3 - Somewhat wrong	4	5 - Not wrong at all
◯	◯	◯	◯	◯

*24. Please indicate how well each of the following statements describes you:

	1 - Does not describe me well	2	3	4	5 - Describes me very well
1. I daydream and fantasize, with some regularity, about things that might happen to me.	◯	◯	◯	◯	◯
2. I often have tender, concerned feelings for people less fortunate than me.	◯	◯	◯	◯	◯
3. I sometimes find it difficult to see things from the "other guy's" point of view.	◯	◯	◯	◯	◯
4. Sometimes I don't feel very sorry for other people when they are having problems.	◯	◯	◯	◯	◯
5. I really get involved with the feelings of the characters in a novel.	◯	◯	◯	◯	◯
6. In emergency situations, I feel apprehensive and ill-at-ease.	◯	◯	◯	◯	◯
7. I am usually objective when I watch a movie or play, and I don't often get completely caught up in it.	◯	◯	◯	◯	◯
8. I try to look at everybody's side of a disagreement before I make a decision.	◯	◯	◯	◯	◯
9. When I see someone being taken advantage of, I feel kind of protective towards them.	◯	◯	◯	◯	◯
10. I sometimes feel helpless when I am in the middle of a very emotional situation.	◯	◯	◯	◯	◯
11. I sometimes try to understand my friends better by imagining how things look from their perspective.	◯	◯	◯	◯	◯
12. Becoming extremely involved in a good book or movie is somewhat rare for me.	◯	◯	◯	◯	◯
13. When I see someone get hurt, I tend to remain calm.	◯	◯	◯	◯	◯

14. Other people's misfortunes do not usually disturb me a great deal.	○	○	○	○	○
15. If I'm sure I'm right about something, I don't waste much time listening to other people's arguments.	○	○	○	○	○
16. After seeing a play or movie, I have felt as though I were one of the characters.	○	○	○	○	○
17. Being in a tense emotional situation scares me.	○	○	○	○	○
18. When I see someone being treated unfairly, I sometimes don't feel very much pity for them.	○	○	○	○	○
19. I am usually pretty effective in dealing with emergencies.	○	○	○	○	○
20. I am often quite touched by things I see happen.	○	○	○	○	○
21. I believe that there are two sides to every question and try to look at them both.	○	○	○	○	○
22. I would describe myself as a pretty soft-hearted person.	○	○	○	○	○
23. When I watch a good movie, I can very easily put myself in the place of a leading character.	○	○	○	○	○
24. I tend to lose control during emergencies.	○	○	○	○	○
25. When I'm upset at someone, I usually try to "put myself in his shoes" for a while.	○	○	○	○	○
26. When I'm reading an interesting story or novel, I imagine how I would feel if the events in the story were happening to me.	○	○	○	○	○
27. When I see someone who badly needs help in an emergency, I go to pieces.	○	○	○	○	○
28. Before criticizing somebody, I try to imagine how I would feel if I were in their place.	○	○	○	○	○

*25. Please indicate how strongly you agree with the following statements.

	Strongly Disagree	Disagree	Slightly Disagree	Slightly Agree	Agree	Strongly Agree
1. I trust my initial feelings about people.	◯	◯	◯	◯	◯	◯
2. I don't like to have to do a lot of thinking.	◯	◯	◯	◯	◯	◯
3. I believe in trusting my hunches.	◯	◯	◯	◯	◯	◯
4. I try to avoid situations that require thinking in depth about something.	◯	◯	◯	◯	◯	◯
5. My initial impressions of people are almost always right.	◯	◯	◯	◯	◯	◯
6. I prefer to do something that challenges my thinking abilities rather than do something that requires little thought.	◯	◯	◯	◯	◯	◯
7. When it comes to trusting people, I can usually rely on my "gut feelings".	◯	◯	◯	◯	◯	◯
8. I prefer complex to simple problems.	◯	◯	◯	◯	◯	◯
9. I can usually feel when a person is right or wrong, even if I can't explain how I know.	◯	◯	◯	◯	◯	◯
10. Thinking hard and for a very long time about something gives me little satisfaction.	◯	◯	◯	◯	◯	◯

General Views on Truthfulness

The next pages of this survey contain questions concerning you general views on truthfulness.

In medical care, it is sometimes the case that a doctor seems to be justified in believing that a better overall outcome can be achieved by refraining from providing patients with certain information.

If the doctor believes that providing a patient with information about their condition is more likely to do more harm than good, to what extent is their decision to omit to tell patients this information:

*26. Praiseworthy?

1 - Not at all praiseworthy	2	3	4	5	6	7 - Very Praiseworthy
○	○	○	○	○	○	○

*27. Moral?

1 - Very Immoral	2	3	4	5	6	7 - Very Moral
○	○	○	○	○	○	○

*28. Blameworthy?

1 - Not at all blameworthy	2	3	4	5	6	7 - Very Blameworthy
○	○	○	○	○	○	○

*29. Outrageous?

1 - Not at all Outrageous	2	3	4	5	6	7 - Extremely Outragepus
○	○	○	○	○	○	○

In some extreme cases, a doctor may only be able to help a patient feel better by deceiving them about their medication.

To what extent is such deception:

***30. Moral?**

1 - Very Immoral	2	3	4	5	6	7 - Very Moral
○	○	○	○	○	○	○

***31. Praiseworthy?**

1 - Not at all praiseworthy	2	3	4	5	6	7 - Very Praiseworthy
○	○	○	○	○	○	○

***32. Blameworthy?**

1 - Not at all blameworthy	2	3	4	5	6	7 - Very Blameworthy
○	○	○	○	○	○	○

***33. Outrageous?**

1 - Not at all Outrageous	2	3	4	5	6	7 - Extremely Outragepus
○	○	○	○	○	○	○

***34. Acceptable?**

1 - Completely Unacceptable	2	3	4	5	6	7 - Absolutely Acceptable
○	○	○	○	○	○	○

Suppose you want to deceive someone and make them believe something that isn't true.

***35. Highlight which of the following you think is worse:**

- a) Providing them with false information.
- b) Giving them true information that is likely to lead them to form the false belief, without telling them other important information that shows it to be false.
- c) Are (a) and (b) morally equivalent?

*36. In general, truthfulness is something that . . .

	Strongly Disagree	Disagree	Slightly Disagree	Slightly Agree	Agree	Strongly Agree
1 - . . . one should not sacrifice, no matter what the benefits.	◯	◯	◯	◯	◯	◯
2 - . . . one should be flexible about if the situation demands it.	◯	◯	◯	◯	◯	◯

Demographic Information

Please provide us with the following details about yourself.

This will not be used to identify you in the data, but will be used to provide us with more information about the people participating in our study.

37. What is your age?

- 18 to 24
- 25 to 34
- 35 to 44
- 45 to 54
- 55 to 64
- 65 to 74
- 75 or older

38. What is your gender?

- Female
- Male

Other (please specify)

39. Do you have a religious affiliation? If yes, please write it.

40. How religious do you consider yourself to be?

1 - Not at all	2	3	4	5	6	7 - Very much
○	○	○	○	○	○	○

41. What is the highest level of education you have completed?

Mechanical Turk Payment Information

Thank you for completing this survey! Please provide the following information for payment purposes on the Mechanical Turk website.

***42. Please provide us with your mechanical turk worker ID (for payment purposes):**

***43. In the comments box below, please make up a completion code that begins with the letters 'JP' followed by any 5 single digit numbers (for example JP19856). You will need to enter the code you write here on the Mechanical Turk Website to receive payment.**
